# Bi-DCNet: Bilateral Network with Dilated Convolutions for Left Ventricle Segmentation

**DOI:** 10.3390/life13041040

**Published:** 2023-04-18

**Authors:** Zi Ye, Yogan Jaya Kumar, Fengyan Song, Guanxi Li, Suyu Zhang

**Affiliations:** 1 School of Artificial Intelligence, Wenzhou Polytechnic, Wenzhou 325035, China; yezi1022@gmail.com; 2 Faculty of Information and Communication Technology, Universiti Teknikal Malaysia Melaka, Melaka 76100, Malaysia; yogan@utem.edu.my; 3 Shanghai Gen Cong Information Technology Co., Ltd., Shanghai 201300, China; songfy@ai-galaxy.com; 4 School of Mechanical and Automotive Engineering, South China University of Technology, Guangzhou 510006, China; liguanxi0809@163.com

**Keywords:** left ventricle segmentation, bilateral network, convolutional neural network, dilated convolution

## Abstract

Left ventricular segmentation is a vital and necessary procedure for assessing cardiac systolic and diastolic function, while echocardiography is an indispensable diagnostic technique that enables cardiac functionality assessment. However, manually labeling the left ventricular region on echocardiography images is time consuming and leads to observer bias. Recent research has demonstrated that deep learning has the capability to employ the segmentation process automatically. However, on the downside, it still ignores the contribution of all semantic information through the segmentation process. This study proposes a deep neural network architecture based on BiSeNet, named Bi-DCNet. This model comprises a spatial path and a context path, with the former responsible for spatial feature (low-level) acquisition and the latter responsible for contextual semantic feature (high-level) exploitation. Moreover, it incorporates feature extraction through the integration of dilated convolutions to achieve a larger receptive field to capture multi-scale information. The EchoNet-Dynamic dataset was utilized to assess the proposed model, and this is the first bilateral-structured network implemented on this large clinical video dataset for accomplishing the segmentation of the left ventricle. As demonstrated by the experimental outcomes, our method obtained 0.9228 and 0.8576 in DSC and IoU, respectively, proving the structure’s effectiveness.

## 1. Introduction

Cardiovascular disease, whose global mortality has been rising, is considered a severe threat to the health of humankind [1]. Accordingly, growing attention has been paid to research and technology pertaining to the improvement of cardiac detection and reduction in the mortality rate associated with these diseases. One of the main concerns and the focus of heart function research and disease diagnosis is the left ventricle (LV). In cardiac functionality research, the left ventricle (LV) constitutes one of the chief concerns and is the diagnostic focus. LV boundary delineation is clinically critical in evaluating cardiac indices, such as the ejection fraction (EF), end-diastolic volume (EDV), and end-systolic volume (ESV) [2].

**Figure 1:**
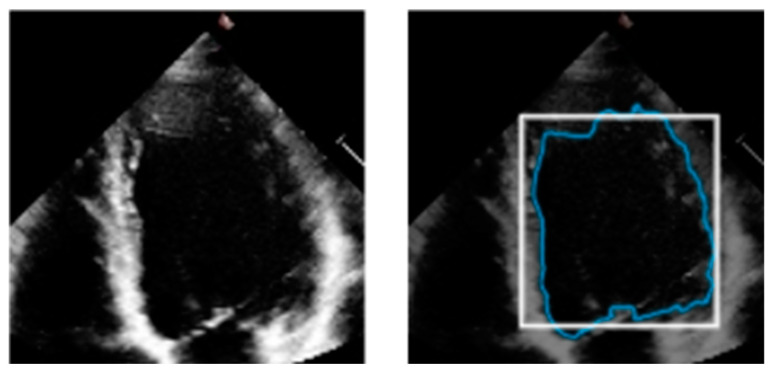
US image (**left**) and its US image with LV boundary (**right**).

Various medical imaging modalities have been utilized to evaluate the LV, and due to improvements in these medical imaging methods, it is now easier to diagnose cardiovascular problems. For example, echocardiography creates cardiac anatomical images using ultrasound (US) waves with high frequencies. Moreover, ultrasound is indispensable for assessing LV function due to its ease of access, exceptional temporal resolution, non-invasiveness, and real-time execution [3]. An example of a two-dimensional US image is shown in Figure 1, wherein the blue track indicates the LV boundary, which is an example output of manual segmentation. A specialist, usually a cardiologist, very meticulously segments the endocardial border of the LV at the end-systolic and end-diastolic phases. This information is then used to offer a quantitative functional examination of the heart in order to identify cardiopathies.

Accurate US segmentation of cardiac anatomy currently relies on sonographers, resulting in tedious jobs reliant on manual competency [4]. In addition, it is challenging for human operation due to low contrast, speckle noise, and poorly defined boundaries [5]. Lately, deep learning (DL) has been introduced as a completely automated technique for processing images, which can help in accurate automatic LV segmentation. We will introduce some mainstream methods and related systems on the EchoNet-Dynamic [6] dataset, which is the largest publicly available set of apical 4-chamber (A4C) echocardiography videos with tracings and cardiac function labels.

In recent years, deep learning has become the most extensively utilized approach for cardiac image segmentation [7]. The adoption of DL in a number of medical fields has generated significant interest in the field of medical image analysis. Ouyang et al. [8] first developed a convolution model to generate frame-level semantic segmentation on the EchoNet-Dynamic dataset. Then, encouraged by the successful application of the transformer structure in the imaging-related task, a lightweight hybrid model integrating the transformer and the CNN architecture was proposed [9]. The highlight of this work is the utilization of shuffled group convolution for redesigning new patch embeddings, which helps diminish the excessive number of parameters.

Different hybrid types of learning models, such as self-supervised and semi-supervised learning, have achieved enormous success in image segmentation [10]. The self-supervised approach, or self-supervision, has proven useful when access to labeled data is limited [11]. A self-supervised contrastive learning method was proposed in the literature to segment the left ventricle when limited annotated images exist [12]. It self-trains by leveraging one portion of the data to predict the other part and generate labels accurately. Another newly revised version of semi-supervised LV segmentation was introduced, which exploits graph signal processing [13]. In this work, instance segmentation and temporal, texture, and statistical feature extraction were required to represent the nodes, followed by graph sampling, where several labeled data were utilized to embed the graph.

Recently, more research focus has been placed on directly segmenting the boundaries of the LV based on time-varying 2D echocardiogram videos, since cardiac movement provides a more detailed description of heart function. For example, based on segmenting the entire cardiac cycle, Puyol-Antón et al. [14] developed an AI-based model for obtaining advanced systolic and diastolic LV function biomarkers. In addition, Chen et al. [15] employed a modified R2 + 1 D ResNet stem to construct a fully automated framework that offered motion tracking apart from joint semantic segmentation in multi-beat echocardiograms. This convolutional network operates sliding windows to accommodate the heart cycle while efficiently encoding spatiotemporal data.

In summary, convolutional neural network (CNN)-based models have remarkably improved LV segmentation on the EchoNet-Dynamic dataset. The evaluations of the segmentation network in the reviewed literature are summarized in Table 1. However, these approaches failed to integrate the spatial features (low-level) with the contextual semantic features (high-level) in an efficient manner.

On the basis of the coarse and fine pathways, a dual-branch network is another method to segment echocardiogram images. A typical bilateral network is made up of a feature fusion module, a context path, and a spatial path. The feature fusion module is used to merge the features learned from both the spatial path and the context path, which are meant for capturing low-level spatial characteristics and high-level context features, respectively. Moreover, few studies have used bilateral architecture to segment echocardiograms, whether in open datasets or other private datasets, according to our research of the literature. Therefore, this paper presents a bilateral network with dilated convolutions, which achieves precise segmentation of the LV in four-chamber echocardiograms based on high- and low-level features. The chief contributions of our methods are as follows:

- Considering low-level and high-level features, we adopted a bilateral-structured network, namely Bi-DCNet, to accurately segment the LV in four-chamber view images. The two branches are responsible for semantic and spatial information, respectively.
- To improve the network’s ability to extract high-level semantic features, dilated convolutions were utilized in the context path, in addition to expanding the receptive field to capture multi-scale information.
- We conducted experiments on EchoNet-Dynamic, a dataset offering 10,030 echocardiogram videos (apical 4-chamber view) with corresponding labeled segmentation tracings of the LV. To the best of the authors’ knowledge, this is the first bilateral-shaped network implemented on this large clinical video dataset to assess cardiac function.

The remainder of the present article is arranged as follows: In Section 2, every module of our current methodology is elaborated. Moreover, the experimental setup and datasets are described, as well as the evaluation matrices. Then, Section 3 summarizes the findings, and Section 4 presents the discussion. Lastly, Section 5 presents the conclusion of this study.

## 2. Materials and Methods

As mentioned above, various techniques have been utilized on this EchoNet-Dynamic for segmenting the LV, yielding desirable results. Encouraged by the effectiveness of DL, the present study put forward a DL methodology for segmenting the LV from echocardiograms. This section will detail the method and how the experiments were conducted.

### 2.1. Methodology

**Figure 2:**
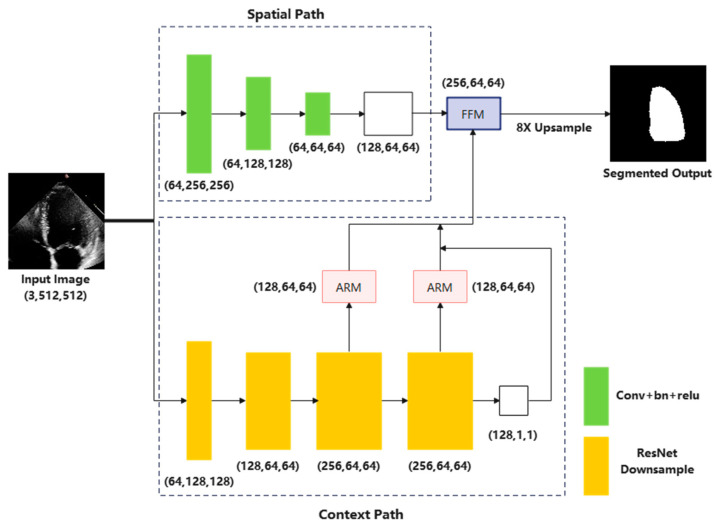
The network architecture of Bi-DCNet.

In this part, we will introduce the proposed Bi-DCNet model, inspired by the classic Bilateral Segmentation Network (BiSeNet) [16], with the following sections: (1) Spatial path; (2) Context Path; (3) Feature Fusion Module; and (4) Loss Function. The complete architecture of Bi-DCNet is shown in Figure 2.

#### 2.1.1. Spatial Path

The spatial path achieves the encoding of abundant spatial information without changing the input image’s spatial size. It adopts three layers, each encompassing a convolution (stride size = 2) and subsequent batch normalization [17] and ReLU [18]. Therefore, apart from generating a feature map whose spatial resolution is 1/8 of the original, this path encodes abundant spatial information while keeping the computational expense low.

#### 2.1.2. Context Path

In order to ensure real-time performance and improve the calculation rate, high-level semantic information is acquired by exploring the lightweight ResNet-18 [19] backbone in the context path. In addition, a global average pooling layer [20] is added to ResNet-18 to offer global context information.

It is general knowledge that during a normal cardiac cycle, the left ventricle (LV) alters both its size and form as a direct result of the contraction and relaxation phases that take place [21]. Hence, we decided to employ dilated convolution [22] with varied dilation rates to address the LV shape variability issue. This permits the extraction of several features correcting for the apparent fluctuation of the LV.

It is also worth mentioning that another advantage of applying dilated convolution layers is leading to a larger sufficient receptive field and achieving great significance for segmentation performance [23]. By using a dilation rate of 2, a 3×3 kernel can reach the receptive field size of a 5×5 kernel, while the network complexity remains the same. To be more specific, for every stage in this path, the dilation rates are (1,1,2,4), and the strides are (1,2,1,1).

**Figure 3:**
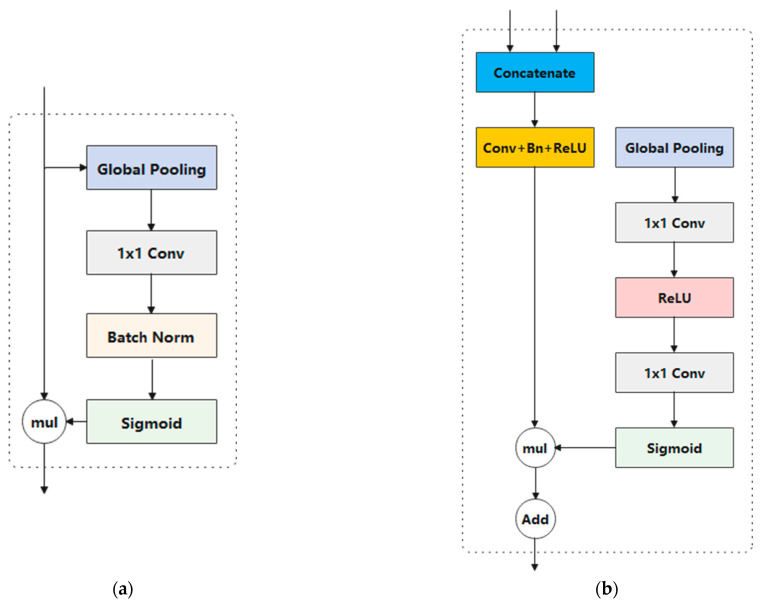
Components of the attention refinement module (**a**) and the feature fusion module (**b**).

Moreover, feature refinement for the last two stages of downsampling was achieved by incorporating an attention refinement module (ARM). As depicted in Figure 3a, the extracted features are initially input into the global pooling layer; then, the computation of an attention vector proceeds for importance reweighting of the features.

#### 2.1.3. Feature Fusion Module

There is a distinction in the level of feature representation between the two feature modules. The spatial path extracts abundant and detailed low-level spatial information, whereas the output feature of the context path includes predominantly high-level semantic information. Degradation of information would therefore result if low-level and high-level features were combined or concatenated directly.

As a result, we fused these features using a particular Feature Fusion Module (FFM). Initial concatenation of the features output from the two paths was followed by balancing the scale via batch normalization. Finally, it was trained to reweight the features for feature selection and combination using a weight vector, such as SENet [24]. The details of the FFM are displayed in Figure 3b.

#### 2.1.4. Loss Function

The loss function plays a crucial role in the DL-based segmentation method. Similar to deep supervision, this approach has one primary loss, along with two auxiliary losses for network supervision. Equation (1) shows that the loss functions are all typical cross-entropy losses.

(1) $$loss=-\frac{1}{N}\underset{i}{\sum }log\frac{exp\left(p_{i}\right)}{\sum_{j}\exp\left(p_{j}\right)}$$

where $p_{i}$ stands for network prediction. As the weighted sum of the two loss types, the overall loss function is formulated as shown in Equation (2).

(2) $$l\left(X;W\right)=l_{p}\left(X;W\right)+\overset{K}{\underset{i=2}{\sum }}\alpha_{i}l_{i}\left(X_{i};W\right)$$

where W refers to the network weight, the value of K is 3, $\alpha_{i}$ is responsible for weight balancing between the two loss types, and $l_{i}$ and $X_{i}$ stand separately for the context path’s loss function and output features, respectively, at the ith stage.

### 2.2. Experiment

The present section elaborates on the dataset exploited for the training and testing of the Bi-DCNet model. Moreover, the hardware, hyperparameters, and evaluation metrics used to analyze the performance of the Bi-DCNet model are described.

#### 2.2.1. Dataset

EchoNet-Dynamic offers apical 4-chamber (A4C) cardiac echocardiograms acquired by Stanford University Hospital, the largest public dataset of its kind. Since the dataset includes 10,030 echocardiogram clips from 10,030 randomly selected patients who underwent echocardiography between 2006 and 2018 as part of clinical care at the hospital, each video represents a distinct individual. In order to locate the video of the apical 4-chamber view, the Digital Imaging and Communications in Medicine (DICOM) files associated with measurements of ventricular volume used to calculate the ejection fraction were extracted in the apical 4-chamber view. Table 2 and Table 3 provide details about the characteristics of the echocardiograms and manufacturers used in this study, respectively.

Among the 10,030 videos it contained, 9989 video samples were finally used after data cleansing. Only the end-diastolic and end-systolic frames were analyzed for each video, and the LV region was annotated by expert cardiologists and sonographers during the routine clinical workflow. Hence, 14,920 of 19,978 images were selected for training, while the rest were used separately for validation (2576 images) and testing (2482 images). Moreover, the end-diastolic and end-systolic frames of identical subjects were assigned to the same groups.

The images were all subjected to appropriate center cropping and resized to 512×512. For data augmentation, random flip with a probability of 0.5 and random rotation in the range of ±25 degrees were used in the training phase.

#### 2.2.2. Network Training

The computer used for performing the entire experiment comprised an Intel(R) Xeon(R) CPU E5-2678 v3 @2.5GHz, 4 GPU NVIDIA RTX 2080TI, and 32G of RAM. During the experiments, the PyTorch and MMSegmentation [25] open-source toolboxes were employed.

In the training phase, stochastic gradient descent (SGD) was used to optimize the network, where the weight decay and momentum were 0.0005 and 0.9, respectively. It is worth noting that a ”poly” learning rate strategy [26] was also applied in which $\eta =\eta_{0}{\left(1-\frac{n}{N}\right)}^{\beta }$, with η and $\eta_{0}=0.0001$ representing separately for learning rate and initial learning rate, respectively, n and N respectively representing the current epoch and total epochs, and the value of β being 0.9.

Random initialization was implemented on all the remaining Bi-DCNet components with the default configuration of PyTorch. Every model was subjected to 160,000 iterations of training, where the batch size was 96. Finally, the best-validated model configuration was chosen to assess the test dataset for model evaluation.

#### 2.2.3. Evaluation Metrics

With the trained models, segmented binary masks were initially created for the test images. Subsequently, the created masks were compared with the ground truth binary mask. The last step was the performance assessment of the proposed model and the comparison of its performance with other semantic segmentation models, where the most popular evaluation matrices were used, namely recall, accuracy, specificity, precision, intersection over union (IoU), and dice similarity coefficient (DSC) [27]. The following are the definitions of each evaluation index:

(3) $$Accuracy=(TP+TN)/\left(TP+FN+TN+FP\right)$$

(4) $$Recall=TP/\left(TP+FN\right)$$

(5) $$Specificity=TN/\left(TN+FP\right)$$

(6) $$Precision=TP/\left(TP+FP\right)$$

(7) $$DSC=2TP/\left(2TP+FN+FP\right)$$

(8) $$IoU=DSC/\left(2-DSC\right)$$

where TP is the number of correctly divided left ventricle pixels, TN is the number of correctly recognized background pixels, FP are the background pixels mistakenly marked into left ventricle pixels, and FN are the left ventricle pixels wrongly marked as background pixels.

## 3. Results

This section presents the findings and experimental outcomes of LV segmentation. The proposed model was assessed for effectiveness against two popular DL segmentation models, U-Net [28] and BiSeNet. During efficacy exploration of the foregoing models, 2,482 test images were used.

### 3.1. Numerical Results

The model with the highest validation accuracy during the training was selected to evaluate the final test results, and the illustrations of peak validation accuracy are shown in Table 4. Our proposed Bi-DCNet model reached a peak validation accuracy of 0.9725 on the iteration of 120,000, the BiSeNet structure had the highest validation value of 0.9704 at the iteration of 160,000, and the U-Net network had the highest validation value of 0.6987 at the iteration of 160,000.

Based on the aforementioned six evaluation matrices, the proposed Bi-DCNet’s segmentation performance is contrasted in Table 5, which details the average values of the six matrices for all the test images.

The DSC and IoU of the proposed model were 0.9228 and 0.8576, respectively, on test images, while the accuracy, recall, precision, and specificity attained were, respectively, 0.9861, 0.9248, 0.9274, and 0.9925. The proposed Bi-DCNet with a bilateral stage outperformed the other segmentation architectures. The Bi-DCNet model achieved feature extraction via ResNet-18, followed by separate processing of spatial and contextual traits so that the extracted information and features could be utilized to their maximum potential. We can also see from the table that the dilated convolutions contributed to the network performance improvement by approximately 0.2% on both the DSC and IoU matrices.

For each model, Table 6 displays the amount of time spent training the segmentation of the LV from the Echo-Dynamic dataset. According to the data in Table 6, the bilateral family networks’ more complex architecture made it necessary for them to train for image segmentation over a significantly longer period of time. The extraction of semantic and spatial information was carried out via the coarse and fine branches of this dual-path network. Moreover, in order to merge feature representations at various levels learned from the dual pathways, several fusion modules were also used [29].

Conversely, the U-shaped network had a significantly shorter training time, at approximately only 16 h. U-Net is a well-known network for segmenting images based entirely on fully convolutional neural networks. Both the network upsampling and downsampling stages employ the same amount of convolutional layer operations. Since the sample layers are interconnected, the features extracted by the downsampling layer can be directly passed to the upsampling layer [30]. However, its performance was not competitive with the proposed network.

### 3.2. Visual Results

**Figure 4:**
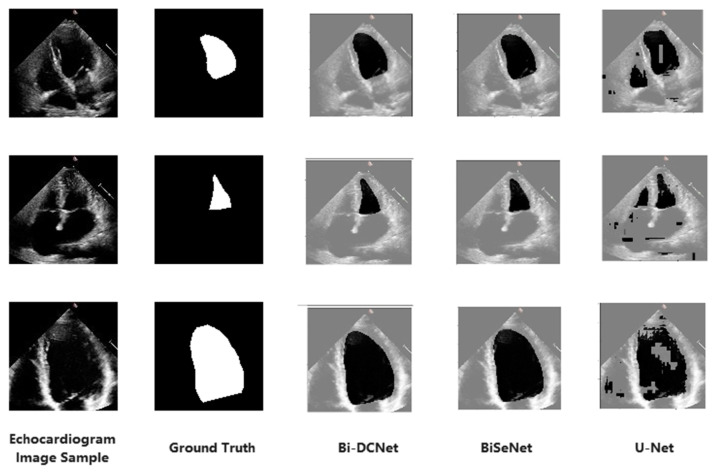
Visual comparisons of the proposed model to other segmentation models.

Figure 4 displays the segmented outputs in visual form for all models. The location of ground truth labels is the leftmost column adjacent to the original echocardiogram images. For the suggested model (Bi-DCNet), BiSeNet, and U-Net, their segmented outputs are also shown in Figure 4’s columns 3, 4, and 5, respectively. Unfortunately, the segmentation accuracy of U-Net is insufficient, primarily because the borderlines are poorly defined, and some background pixels are also included as target objects.

However, the Bi-DCNet and BiSeNet borderlines are rather precise. In order to preserve the spatial information of the image and produce a high-resolution feature map, both structures created three convolutional layers with a short step size, using a more significant number of channels and a shallower network. On the other side, channel attention-based ARM selected the discriminative high-level semantic features from the last two steps of the context path. This led to the much better visual results of Bi-DCNet and BiSeNet, illustrating that the bilateral structures had better accuracy regarding left ventricle representation than the U-shaped structure.

## 4. Discussion

This work suggests a bilateral segmentation model and evaluates its performance against U-Net and BiSeNet. We adopted a bilateral structured network for LV segmentation in echocardiography, where conventional segmentation approaches are not satisfactory due to low signal-to-noise ratios, fluctuating levels of speckle noise, and the existence of shadowing in the ultrasound. According to a literature review, this is the first time a bilateral structure was used on the EchoNet-Dynamic dataset, and a considerable improvement in segmentation accuracy was achieved. Moreover, since the left ventricle’s area and volume vary significantly throughout the cardiac cycle due to constant contraction and expansion, dilated convolution with varied dilation rates was first integrated into the context module in order to capture multi-scale feature information.

The provided model demonstrated the highest performance levels regarding DSC, IoU, accuracy, precision, and specificity, as indicated in Table 5. The suggested method used the RestNet-18 model to extract features, which were then separately processed to maximize the use of the information and features.

On the other hand, the benefit of employing dilatation convolution over conventional convolution processes is the possibility of achieving more extensive receptive fields while preserving the same feature resolution and reducing the number of parameters [31]. Additionally, the model is better able to comprehend information about the whole context—for instance, the left ventricle’s shape changes throughout the cardiac cycle [32]. We employed dilated convolution with varied dilation rates in the context detail module to capture multi-scale feature information in order to more precisely separate the left ventricle, with its various sizes and appearances. This can increase the left ventricle’s segmentation precision during echocardiography.

**Figure 5:**
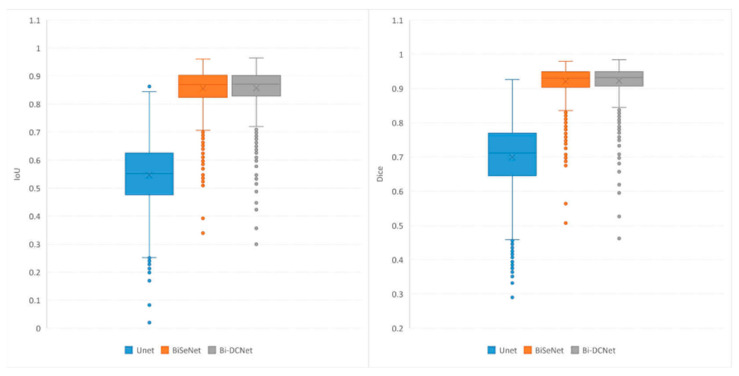
Boxplots of IoU (**left**) and Dice (**right**).

In addition to the numerical and visual results, statistical outcomes were also offered for the evaluation metrics. All test images’ evaluation metric distributions were analyzed and contrasted using a boxplot. Figure 5 (left) is a boxplot depicting the IoU values for the test images. The two bilateral networks clearly surpassed the U-Net model in terms of maximum values, minimum values, and medians, as well as the highest and lowest quartiles. Moreover, the proposed Bi-DCNet exhibited slightly higher performance than the classic Bi-DCNet, indicating that the dilated convolutions helped capture a larger global context.

Boxplots for DSC values are depicted in Figure 5 (right). As inferred from the figure, the behavior of the proposed model for DSC metrics is quite similar to that of IoU. In addition, the reduced skewness of the boxplot for the proposed Bi-DCNet, in contrast to those of the other models, demonstrates the superior performance of the proposed network.

**Figure 6:**
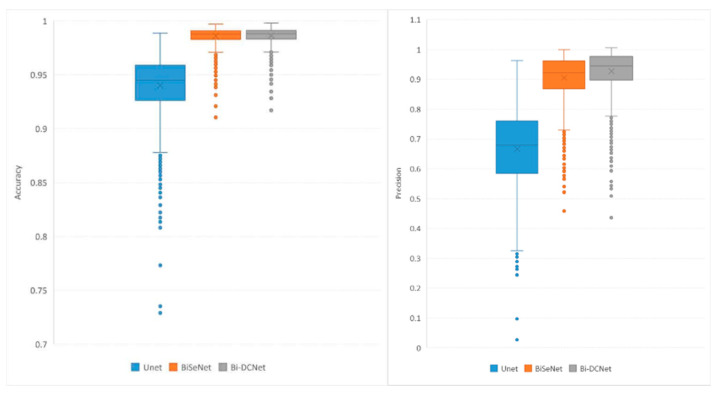
Boxplots of Accuracy (**left**) and Precision (**right**).

**Figure 7:**
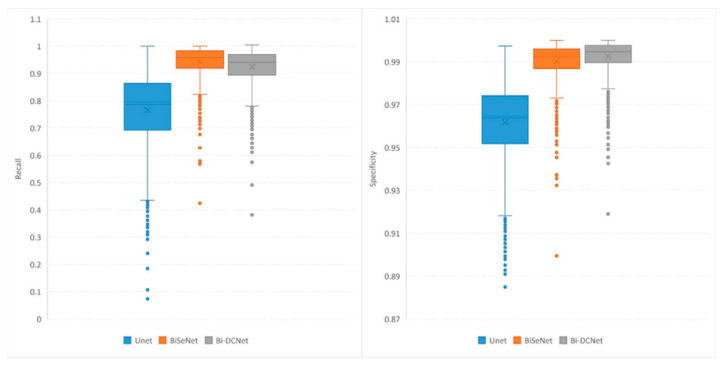
Boxplots of Recall (**left**) and Specificity (**right**).

Boxplots for recall, precision, accuracy, and specificity are depicted in Figure 6 and Figure 7, which provide additional evidence that performance improvements were achieved when both bilateral structure and dilated convolution were employed to collect local and global information.

## 5. Conclusions

This article presents Bi-DCNet, a DL network that exploits bilateral structures to achieve completely automatic segmentation of the left ventricle in four-chamber view echocardiograms. Our proposed model consists of a spatial path responsible for low-level feature acquisition, a context path for high-level feature acquisition, and a fusion module that enables efficient integration of features captured by the preceding two paths.

It is essential to note that the use of dilated convolutions, which offers a broad receptive field size for a more global context, is most significant in improving segmentation performance. Although the fact that dilated convolution provides a large receptive field size, the segmentation mask formed by a single dilation rate throughout the segmentation process does not cover all semantic strengths. To successfully capture multi-scale objects, we applied multiple dilated convolutions with varying dilation rates in the bottleneck layer, a particular dilation method extensively used in the field of medical imaging.

The proposed model demonstrated impressive performance, with a DSC of 0.9228 and an IoU of 0.8576, outperforming the well-known deep learning techniques U-Net and BiSeNet. Despite the fact that our proposed model Bi-DCNet did not noticeably outperform BiSeNet, it is crucial to highlight that this manuscript applies a bilateral structure to the EchoNet-Dynamic dataset for the first time, with excellent results. Given that BiSeNet’s output is already satisfactory (0.9207), we applied dilated convolution on this basis to account for the uncertainty of the size of the left ventricle. Although the improvement (approximately 0.2%) is not immediately apparent, it is clear that dilated convolution accumulated to some degree. We hope to use this technique in the future for tasks such as automatic measuring and landmark recognition in medical image analysis.

## Figures and Tables

**Table 1:** Comparative evaluations of the automated LV segmentation network from previous works.

References	Dice Similarity Coefficient
Ouyang et al. [8]	0.9270
Deng et al. [9]	0.9164
Saeed et al. [12]	0.9252
El rai et al. [13]	0.9389
Puyol-Antón et al. [14]	0.9350
Chen et al. [15]	0.9440

**Table 2:** Characteristics of the echocardiograms used in this research.

Age (Years)	Sex (% Female)
57 ± 17	55

**Table 3:** Characteristics of the manufacturers used in this research.

Philips Medical Systems ie33	Acuson Sequoia	Philips Medical Systems EPIQ 7C	GE Vingmed Ultrasound Vivid E9	Philips Medical Systems HD15	GE Vingmed Ultrasound Vivid E95	GE Vingmed Ultrasound Vivid i
44%	14%	14%	11%	11%	4%	2%

**Table 4:** Performance of different models on peak validation accuracy.

Models	Peak Validation Accuracy	Iteration
Bi-DCNet	0.9725	120,000
BiSeNet	0.9704	160,000
U-Net	0.6987	160,000

**Table 5:** Mean and standard deviation values of evaluation metrics.

Models	DSC	IoU	Accuracy	Recall	Precision	Specificity
Bi-DCNet	0.9228 ± 0.0416	0.8576 ± 0.0660	0.9861 ± 0.0076	0.9248 ± 0.0630	0.9274 ± 0.0686	0.9925 ± 0.0074
BiSeNet	0.9207 ± 0.0425	0.8558 ± 0.0678	0.9858 ± 0.0076	0.9433 ± 0.0547	0.9056 ± 0.0750	0.9904 ± 0.0082
U-Net	0.6992 ± 0.0997	0.5461 ± 0.1129	0.9401 ± 0.0272	0.7664 ± 0.1314	0.6665 ± 0.1323	0.9617 ± 0.0173

**Table 6:** Representations of the timing information of the proposed model and other well-known segmentation models.

Models	Training Time
Bi-DCNet	2 days and 19 h
BiSeNet	2 days and 18 h
U-Net	16 h

## Data Availability

The EchoNet-Dynamic dataset is publicly available at https://echonet.github.io/dynamic/ (accessed on 5 March 2023).
